# High-Accuracy Positioning Based on Pseudo-Ranges: Integrated Difference and Performance Analysis of the Loran System

**DOI:** 10.3390/s20164436

**Published:** 2020-08-08

**Authors:** Baorong Yan, Yun Li, Wei Guo, Yu Hua

**Affiliations:** National Time Service Center, Chinese Academy of Sciences, Xi’an 710600, China; liyun@ntsc.ac.cn (Y.L.); guowei@ntsc.ac.cn (W.G.); hy@ntsc.ac.cn (Y.H.)

**Keywords:** Loran, position, geometrical dilution of precision, accuracy, difference method

## Abstract

The Long Range Navigation (Loran) system as a backup of the Global Navigation Satellite System (GNSS) is a good choice. The dominant deterioration factors of position accuracy are the pseudo-range measurement errors and the geometric dilution of precision (GDOP). This paper focuses on the algorithm integrated difference with pseudo-ranges to improve the position accuracy. Firstly, the theoretical prediction of propagation delay and raw measurement are compared. The results show that the measured pseudo-range consists of a constant term and a temporal term, which reflect the propagation situation along the true path. Secondly, a position solution algorithm based on a pseudo-range and difference is presented, exceeding the limit of a single chain. Finally, some simulation tests are implemented utilizing the new proposed position algorithm to verify the differential performance. This method can reduce the GDOP conveniently through increasing the number of transmitters. In view of the amplitude and characteristics of errors in measurement, systematic error and random noise are distinguished and discussed. The absolute accuracy responds to the pseudo-range bias that is different from geometric distance and repeatable accuracy is mainly influenced by random noise. The difference method can improve the absolute accuracy via the correction degree without changing the geometry of the transmitters.

## 1. Introduction

Accurate position is a significant topic that should be intensively studied. Generally, it is easy to get the required accuracy using the Global Navigation Satellite System (GNSS). However, the vulnerability of GNSS to unintentional and intentional interference signals can be found frequently nowadays [1,2,3]. For national security and economic effectiveness, a reliable and complementary navigation system is needed desperately [4]. The suitability of the Loran for a backup navigation system has been evaluated and reported [5]. Since then, as a leading role, the United States Coast Guard (USCG) has tried to improve the performance of the Loran system dramatically by the modernization of equipment [6], which mainly included the improvement of transmitters and reference clocks. The improved effect and application are also very obvious [7]. Besides the US, the United Kingdom (UK), South Korea and other countries have also introduced much research on alternatives to the GNSS backup system [8,9,10,11]. They all identified Loran as the terrestrial radio navigation system that has the potential to fulfil those requirements. China has also carried out relevant research [12,13,14]. Meanwhile, China’s major national infrastructure project for developing terrestrial Loran began recently. Three new transmitters using the Loran-C mechanism will be built in the west region of China, which will construct a full coverage timing and positioning network. In order to meet the stated performance [15] as a backup, many researchers and organizations mostly focus on the correction of the Additional Secondary Factor (ASF), which still plays an importance role in position and timing [16,17,18,19,20,21]. The results show that ASF varies with time and can be modified by the difference method, which will be used to improve position accuracy. Nevertheless, the effect of difference is not involved or discussed [22]. So, it is necessary to research the different position solutions and performance to upgrade the position accuracy. 

As we all know, geometric distance applied to position can be derived by the product of propagation delay and velocity when the propagation path is smooth and homogeneous. When the propagation distance between the transmitter and receiver is known, the Loran signal propagates along the surface of the Earth, whose velocity is slowed by the atmosphere and the surface dielectric properties of the Earth [19,21]. Based on this, a model predicting propagation delay based on relative dielectric and equivalent conductivity is developed, which contains three main components: Primary Factor (PF), Secondary Factor (SF) and ASF [16,23,24]. However, weather experienced along the propagation path influences the refractive of the atmosphere and the ground conductivity and then cause variations in the propagation delay [24,25,26,27,28]. In other words, propagation delay is not constant and changes with time, so it is difficult to calculate the delay of the Loran signal instantaneously. In some work, researchers have divided propagation delay into spatial and temporal components based on actual measurements, which correspond to a pseudo-range via velocity. Some studies about the impact of the temporal component on Loran position, especially on aviation Non-Precision Approaches (NPAs) and maritime Harbor Entrance and Approach (HEA), have also been done [29]. In fact, systematic error and random noise are involved in the space and temporal components. The influence of observation error on position and the influence of processing on error mitigation need to be studied in detail. 

This paper focuses on a new position method of the Loran system, based on measurement pseudo-range combined difference. The performance of this way via position accuracy is discussed. Before this, the distinction between predicted propagation delay and the measurement one is presented. A fit formula and statistical calculation about the measured propagation delay are illustrated, which reflect the characteristics of delay and pseudo-range variations over time. Accounting for the features of pseudo-ranges, some simple tests using new algorithms are simulated. Firstly, a comparison of the Geometric Dilution of Precision (GDOP) between three and four sites is considered, showing that the new method can easily decrease the GDOP. Then, position accuracy is presented in order to estimate the difference performance with the impact of observation error, including SF, ASF and random noise.

## 2. Pseudo-range and Position Solution Method

### 2.1. Comparion between Predicted Propagation Delay and Measurement

#### 2.1.1. Predicted Propagation Delay

**Figure 1:**
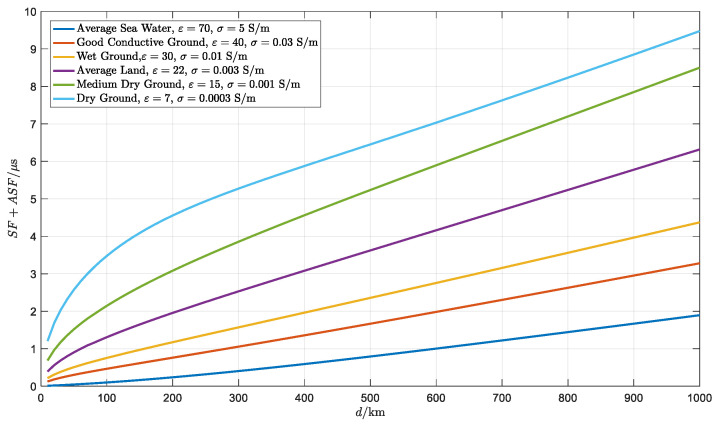
The trend of SF+ASF via distance.

Generally, it is important to convert the propagation delay to the ground truth range in positioning [16]. If the truth range is known, the propagation delay is derived from the distance divided by the speed of light in a vacuum. However, the Loran signal propagates out radially from the transmitter by groundwave, traveling parallel to the surface of the Earth. So, it does not travel at the free space speed of light, rather, it is slowed by the atmosphere and the surface of the Earth. The groundwave accumulates a delay compared to the expected propagation time over the same distance in free space. This delay is the accumulation of three theoretical components or factors [16,17,18,19,23,24,25]. One part is the PF, which is the time delay of the long wave signal propagation through the atmosphere. The second part is the seawater SF, which is the additional delay due to the signal traveling over the sea. The last one is the ASF, the additional delay due to the signal traveling over the land. The specific formula can be seen in Equation (1).

(1) $$T_{p}=PF+SF+ASF$$

where $PF=\frac{R_{d}*n_{s}}{C}$, the light speed C in the vacuum is 299,792,458 m/s and $R_{d}$ is the distance of signal propagation between the transmitter and the receiver. The refractive index of the atmosphere $n_{s}$, assumed as a constant, means that the speed of the signal is a fraction lower than the speed of light in a vacuum. At present, it is difficult to distinguish the SF and the ASF, so SF is often incorporated with ASF as a function of distance $R_{d}$, carrier frequency f, relative dielectric $\varepsilon_{r}$ and equivalent conductivity $\sigma_{e}$, as shown in Equation (2):

(2) $$SF+ASF=\frac{argW\left(f,R_{d},\varepsilon_{r},\sigma_{e}\right)}{\omega }$$

where ω=2πf corresponds to the Loran carrier frequency of 100 kHz and argW is the phase of signal attenuation function W about the Loran. It is known that the value of SF+ASF is a smaller modified term comparing to PF [30]. According to the parameters in [30], SF+ASF via distance for each type of ground is given in Figure 1. When the propagation distance increases the SF+ASF also increases. At a constant distance, SF+ASF is the least for average sea water. 

In the above model, the total propagation delay is calculated using some simplification, similarly to the result of Brunavs’ formula [22]. The result of the calculation is constant if the ground truth distance is known and the dielectric constant with equivalent conductivity is determined. At this level, the effect of climate and weather is not taken into account, or of ground elevation on the index of refraction of the atmosphere on the surface of the ground. Meanwhile, it is not enough to consider the dielectric and equivalent conductivity changes due to freezing rain and snowy weather. These variations are often lumped together for the user. Real-time factors, such as climate and weather, will result in a distinct difference between predicted value and true propagation delay. That is to say, the total propagation delay is not accurate, although it is possible to predict it from Equations (1) and (2).

#### 2.1.2. Measured Propagation Delay and Assessment

In order to describe the true value, a modified formula is presented:

(3) $$T_{p}(t)=PF+SF+ASF+\Delta ASF\left(t\right)$$

where ΔASF(t) accounts for all of the time varying aspects, reflecting the impact of various real-time factors. In other words, ΔASF(t) depends on the weather and ground conductivity along the propagation path over time. Any changes in climate and the conductivity would change ΔASF(t). If the receiver position is not accurate or the propagation path is irregular, the fluctuations would also be contained in ΔASF(t). Similarly, we also divide the propagation delay into two parts: a spatial component $T_{p}$, which is the constant part of $T_{p}\left(t\right)$, and a temporal component ΔASF(t).

**Figure 2:**
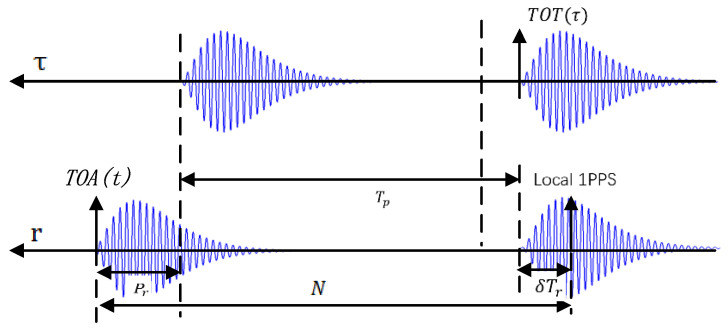
The schematic diagram of propagation delay.

A better way to get propagation delay is to measure it directly. For this purpose, a Loran receiver is used. The measured propagation delay N is the difference between the Time of Arrival (TOA) and Time of Transmission (TOT) of Loran pulses coherently modulated on the carrier ground wave. The measurement principle is shown in Figure 2.

τ in TOT(τ) represents the transmitter’s frame and r in TOA(r) is the receiver time frame. The propagation delay N between receiver and transmitter is defined by Equation (4) below:

(4) $$N=TOA\left(r\right)-TOT\left(\tau \right)=T_{p}+\Delta ASF\left(t\right)+\delta T_{r}+P_{r}+\xi \left(t\right)$$

where $\delta T_{r}$ and $P_{r}$ are the clock bias and the internal processing delays of the receiver, respectively, and ξ(t) represents the random residual error due to the unavoidable noise in artificial measurement. In the Loran frequency band, the dominant source of noise is atmospheric noise, which is caused by lighting discharges and man-made noise and local interference from, for example, switch-mode power supplies. Other sources of noise may include transmitter pulse timing jitter or receiver-related noise [31]. It is likely the accuracy of N itself is a function of t, consisting of a constant part $N^{\prime }$ (i.e., $N^{\prime }=T_{p}+\delta T_{r}+P_{r}$) different from $T_{p}$ and a temporal part ΔASF(t)+ξ(t).

**Figure 3:**
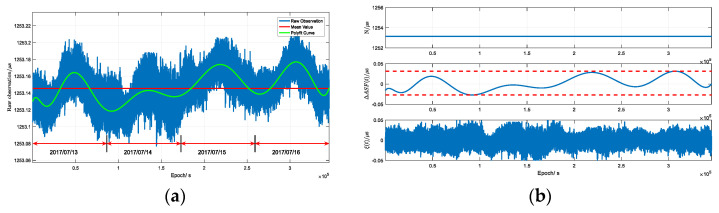
The schematic diagram of measured propagation delay and decomposition. (**a**) Raw measurement; (**b**) Decomposition of propagation delay.

Figure 3 presents a raw measurement of N in a fixed position (109.7503° E, 38.2503° N) for four days, receiving the signal of a BPL transmitter (109.5431° E, 34.9486° N) in Pu Cheng (PC, a city in Shaanxi Province, China). Here, abnormal measurements are removed from the results in advance. The propagation environment is rather homogeneous. Obviously, N varies via time, as seen from the blue line in Figure 3a. A mean value is also plotted by the red line, giving the constant part of N. The average value of the observation is 1253.145 µs, while the predicted constant $T_{p}$ is 1223.860 µs based on the parameters of average land [30] using Equations (1) and (2). Depending upon the navigation application, the difference originating from the effect of $\delta T_{r}+P_{r}$ is negligible, because each transmitter is synchronized and the user receiver clock bias is common. Due to the influence of random noise on raw measurements, polynomial fitting is used to derive the trend of observation, seen as the green curve in Figure 3a.

**Figure 4:**
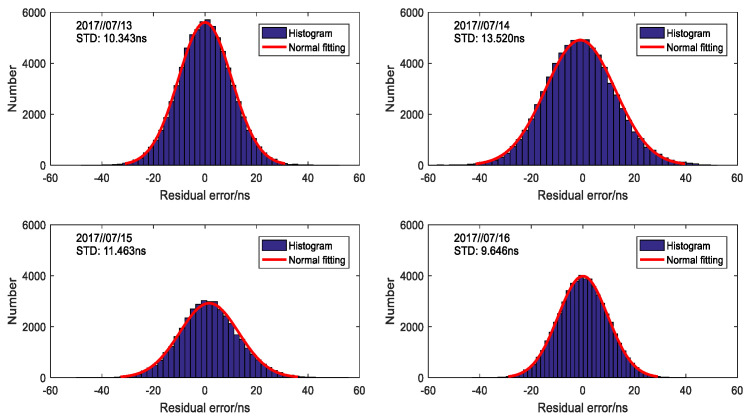
Character of observation noise in propagation delay.

In order to compare the amplitude of each component, Figure 3b gives the decomposition of $N^{\prime }$, ΔASF(t) and ξ(t). It is clear that $N^{\prime }$, the mean value over four days, is the determinant of position and ΔASF(t) is much smaller than the former. However, its variation as time advanced is no more than 30 ns no matter whether it increased or decreased. The amplitude of ξ(t) is similar to ΔASF(t), whose histogram of each day is plotted in Figure 4. The standard deviations (STDs) of calculation for ξ(t) are 10.343 ns, 13.521 ns, 11.463 ns and 9.647 ns, respectively. Besides, normal distribution fitting curves with the same STDs are also presented, which are used to explain the characteristic of ξ(t).

### 2.2. Position Solution Method Integrated Difference

**Figure 5:**
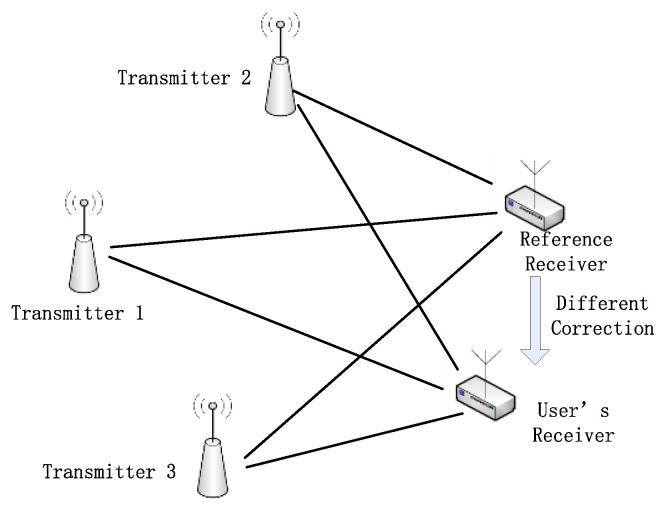
A scheme of the difference correction system.

The difference method is usually used to correct common errors between the user and referent station. A scheme of the difference correction system in this paper is shown in Figure 5. 

Based on the above illustration, the procedure using difference is:

- Consistency test of reference station receiver and user receiver, to determine the processing delay deviation between two receivers.
- Identify the accuracy position of a reference station and get the reference station’s pseudo-range, then derive the difference correction based on the accurate location.
- Obtain pseudo-range observations from at least three transmitters.
- Calculate the correction of each pseudo-range, then use the least squares solution to find the location.

The details are listed in the following part. The measured propagation delays between the transmitting stations and receiver are converted into pseudo-ranges by multiplying the speed. A pseudo-range’s equation for referent station is given, based on the delay relationship, which is used to calculate the difference correction:

(5) $$N_{i}\left(t\right)C=n_{s}R_{di}+C\left(SF_{i}+ASF_{i}+\Delta ASF_{i}\left(t\right)\right)+C\left(\delta T_{r}+P_{r}\right)+C\xi_{i}\left(t\right).$$

where i corresponds to the ith transmitter and i=1,2,3… Other parameters have the same meaning as above. Here, it is necessary that the local clock of the reference station is consistent with the time system of the transmitter or that the time deviation is known and all the transmitters in one chain are synchronized or the inherent difference is known in advance. The relative difference correction, removing $\xi_{i}\left(t\right)$, is expressed as:

(6) $$C\left(SF_{i}+ASF_{i}+\Delta ASF_{i}\left(t\right)\right)=N_{i}\left(t\right)C-n_{s}R_{di}-C\left(\delta T_{r}+P_{r}\right)$$

In the previous work, the database of differential correction is necessary, which cost a large number of measurements [19]. In this paper, according to the analysis of $SF_{i}+ASF_{i}$ in Section 2.1.1 and the amplitude of $\Delta ASF_{i}\left(t\right)$ in Section 2.1.2, differential correction for user position is derived depended on distance relation. A simple data map around the referent station is calculated. The pseudo-range integrated difference equation for the user is given as:

(7) $$N_{i}\left(t\right)C-C\left(SF_{i}+ASF_{i}+\Delta ASF_{i}\left(t\right)\right)=n_{s}R_{di}+C\left(\delta T_{r}+P_{r}\right)+C\xi_{i}\left(t\right)$$

where $C\left(SF_{i}+ASF_{i}+\Delta ASF_{i}\left(t\right)\right)$ on the left side of Equation (7) is the correction corresponding to different transmitters. It is noted that the environmental features of the path from the transmitter to the reference station are similar to those of the path to the user’s receiver. If the paths are very different, the difference method is not suitable. 

In the geodetic coordinate system, the Loran system transmitter’s position $\left(\varphi_{i},\lambda_{i}\right)$ is known. The receiver position (φ,λ), $R_{di}$ and $\delta T_{r}+P_{r}$ are three unknown parameters to be sought. It is necessary to measure three pseudo-ranges used to at least find a position. The algorithm is similar to the GNSS pseudo-range solution based on all available measurements using least squares. Differing from the former algorithm [22], another processing method for the distance between the transmitter and the user is presented in this paper, and details can be seen in Appendix A. With the help of matrix inversion, the main linearized observation equation is thus given by:

(8) A·X=B

where A is the design matrix, X is the unknown quantity and B is the observation vector eliminating the difference correction. The least squares solution is derived as:

(9) $$X={\left(A^{T}\cdot A\right)}^{-1}\cdot A^{T}\cdot B$$

The assumed position $P_{0}\left(\varphi_{0},\lambda_{0},T_{u0}\right)$ is updated by the increment $\left(\Delta \varphi ,\Delta \lambda ,\Delta T_{u}\right)$ and the process is repeated. If the new update is less than 1mm, then no further iterations are considered necessary and we arrive at a “best” Loran fix. 

## 3. Results and Discussion

Raw measurements of propagation delay or pseudo-range contain a number of biases and errors. In order to illustrate the practicability of the pseudo-range method, some detailed simulation tests are discussed in the following parts. The simulation environment is a seawater path, which is partly homogeneous. Another propagation type is considered in the Discussion. 

### 3.1. Geometric Dilution of Precision

In the Loran positioning system, the GDOP between the receiver and the transmitters is a dominant deterioration factor of positioning accuracy. The impact of geometry on the accuracy performance of a ranging system is well understood [32]. An effective method for improving position accuracy is to optimize the geometrical placement of transmitters, thereby decreasing the GDOP. On the other hand, another way to improve performance is by increasing number of stations involved in the position solution when the transmitter station’s location has been confirmed, However, it is difficult to diminish the GDOP using traditional hyperbolic navigation position fixing due to chain restrictions. The pseudo-range method proposed in this paper is convenient to progress two chains or more, not limiting the number of transmitters. As an example, two chains on the east coast of China are used to analyze the GDOP. The details are listed in Table 1. 

**Figure 6:**
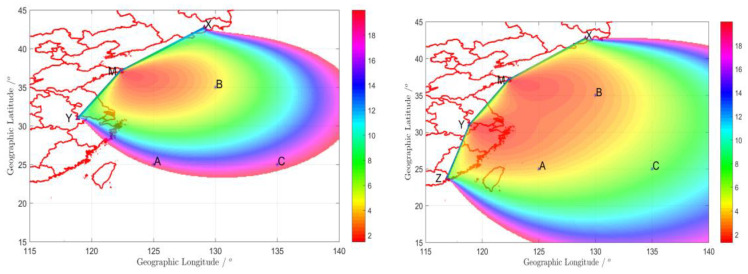
The GDOP of user based on azimuth, the left one is for three sites and the right one is for four sites.

The GDOP is calculated for three and four sites, respectively, using the geodesic and its azimuth, shown in Figure 6. An area with GDOP ≤ 20 is presented. The left one is for three sites (M, X, Y) and the right one is for four sites (M, X, Y, Z). It is obvious that the coverage area of the three sites is smaller than for the four sites. That is to say, the value of the GDOP for the four sites is smaller than for the three sites on fixed points. Table 2 lists three points within coverage, arbitrarily including their coordinates and corresponding results, which are for later analysis.

### 3.2. Position Accuracy

When referring to the accuracy of a positioning system, it is necessary to distinguish its absolute accuracy and repeatable accuracy. According the Loran-C User Handbook [33], the absolute accuracy is defined as the accuracy of a position with respect to the geographic or geodetic coordinates of the Earth. The repeatable accuracy, then, is the accuracy with which a user can return to a position whose coordinates have been measured at a previous time with the same navigational system [31,33]. The true test of the position method is the position accuracy between solutions in a receiver and true fix, where the absolute accuracy and repeatable accuracy are contained.

Before discussion, a specification of some simulation parameters is illustrated in Table 3. Three preferential user sites within coverage range are presented, with coordinates the same as in Table 2. The distances between the test site and each transmitter using Equations (A2) and (A3) in the Appendix A, as well as PF, are also given. Similarly, the predicted value of SF and ASF, assuming all-seawater paths according to Equation (2), are calculated and are presented in Table 3. Based on the former two items, the pseudo-ranges not containing time variations and clock biases are simulated, as seen in Table 3. Additionally, four stations are considered regardless of the number of participants. Here, the different correction progress is ignored and the performance of difference is stressed.

#### 3.2.1. Position without Difference Correction

**Figure 7:**
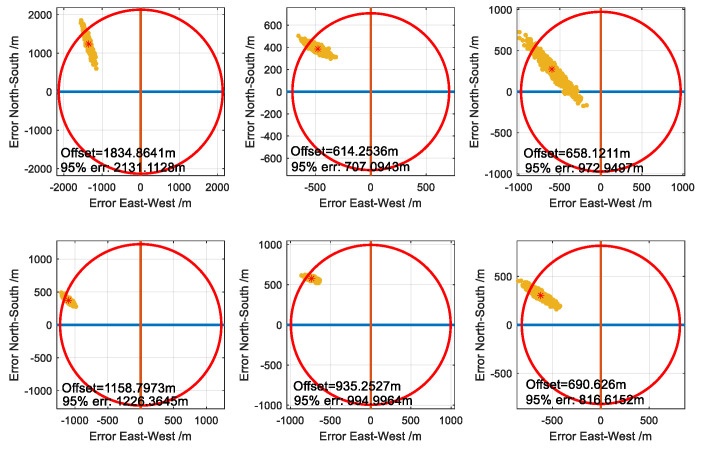
Scatter plot of position accuracy effected by SF + ASF and random noise. The upper three subgraphs are for three transmitters and the bottom three subgraphs are for four sites.

The method without difference is tested. Based on the observation characteristics of the propagation delay referred to in Section 2.1.2, the pseudo-range consists of the distance between the user and transmitter, SF and ASF. Here, the temporal ΔASF is ignored due to a small amplitude. Meanwhile, random noise with an STD of 50ns (i.e., about 15m, which is smaller than the typical value) is added to sampling point 1,000 of each pseudo-range. Actually, the range of noise determined by the signal-to-noise ratio (SNR) of the received signal is temporarily not considered here. All terms in the pseudo-range are considered as measurement biases besides distance related to position. Figure 7 gives the scatter position error using the algorithm proposed in Section 2.2. The longitude and latitude error are represented by the horizontal and vertical axis, respectively. The upper three subgraphs, with respect to the user’s position A, B, C, are the results of the three sites composed of M, X, Y and the bottom three subgraphs are the results of the four sites composed of M, X, Y, Z. The red star is the statistical average corresponding to the case without random noise. A significant absolute offset of position emerges due to the impact of SF and ASF. It implys that the pseudo-range suffers from large measurement biases resulting in absolute accuracy in the order of hundreds of meters or more, seen in the 95% error radius. The error’s directional components, along longitude and latitude in the form of a statistical average, are listed in Table 4, where the STDs of each component represents the repeatable accuracy. Comparing the upper three subgraphs and the bottom one, the repeatable accuracy is improved because the GDOP for the four sites is smaller than for the three sites, that is, a smaller GDOP suppresses the influence of random noise. However, absolute accuracy does not always improve because it also depends on observation error.

#### 3.2.2. Position with Difference

**Figure 8:**
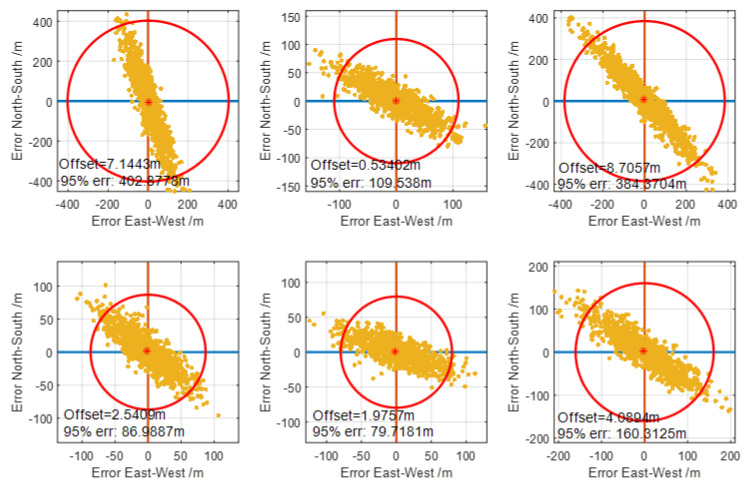
Scatter plot of position accuracy with complete difference, only including random noise in the pseudo-range. The upper three subgraphs are for three transmitters and the bottom three subgraphs are for four cases.

Based on the results of Section 3.2.1, the difference correction is used to mitigate the influence of SF and ASF to obtain the actual distance between transmitter and receiver. For example, the amplitude of SF and ASF is reduced to 70%, that is to say, 30% of the bias in the pseudo-range is corrected. The results are presented in Table 4. Similarly, 70% of biases are corrected and are listed in Table 4, too. It shows that the latitude and longitude direction’s positioning average error decreased proportionally and gradually with the increase in correction. Compared with no difference, the latitude average error is reduced to 873.035 m and 370.236 m, respectively, corresponding to 30% and 70% correction for the A site. However, the STD is almost unchanged because the random noise is not corrected. Ideally, difference would correct all the system errors, implying that there is no SF or ASF in the observations. The absolute error is almost nearly zero for both three and four transmitters, as seen in Table 4. Figure 8 presents the scatter plot of position error by the influence of random noise only, using the algorithm proposed in Section 2.2 with difference. The longitude and latitude errors are represented by the horizontal and vertical axis, respectively. The upper three subgraphs are the results of three sites, composed of M, X, Y. The bottom three subgraphs are the results of four sites, composed of M, X, Y, Z. There is almost no position bias from the statistical mean results, which means a good absolute accuracy performance. The 95% error radius for the B site is smaller than for the A and C sites due to its smaller GDOP. Comparing the upper and bottom subgraphs, the 95% error radius decreases for the bottom case, which also responds to the decreased GDOP for the four transmitters. In a practical application, the difference correction is not always or necessarily proportional, so the correction effect based on arbitrary correction is not improved at all times.

#### 3.2.3. Discussion

In Section 3.2.1 and Section 3.2.2 of the paper, the main influence of SF+ASF and random noise on position accuracy is discussed. The effect of ΔASF with a smaller amplitude is omitted because its influence is similar to SF+ASF, which is system bias corresponding to position. It is shown that difference correction can calibrate the system error only, improving the absolute accuracy. However, if the correction scale of the system error in the observed pseudo-range is inconsistent, i.e., all the observation is non-weighted, then a process method considering unequal precision is very necessary. The least squares solution equation with weight could be expressed as follows:

(10) $$Z={\left(A^{T}\cdot W\cdot A\right)}^{-1}\cdot A^{T}\cdot W\cdot b$$

Naturally, it is important to determine the weighting matrix. One method is based on the SNR of each signal, seen reference [19] for details. As for random noise, it is eliminated significantly by filtering or smoothing the pseudo-range. The repeatable accuracy is improved using a pseudo-range after filtering, such as the Kalman filtering progress. When the proposed pseudo-range method is used, the user receiver needed to obtain three transmitter signals at least. The position of the new transmitter and geometric placement must be considered before using this new method in order to ensure signal reception. 

In the above discussion, the simulation path is seawater, where SF+ASF is the smallest one of all the ground types shown in Figure 1. Clearly, in another propagation type, such as average land, the absolute position bias will be much larger than that for seawater. The difference method is necessary in order to get a higher absolute accuracy. Compared with the actual difference, the degree of correction depends on the correlation of the two paths. One is the path between the transmitter and the reference station. Another one is the path between the same transmitter and the user receiver. A more complicated situation is that the degree of correction is different for all transmitters. A more detailed analysis should be considered in future work.

## 4. Conclusions

In this paper, a theoretical predicted propagation delay of the Loran signal and influential factors are discussed. Moreover, the direct measurement of propagation delay between user and transmitter are illustrated. Both theoretical analysis and measured results showed that propagation delay could be divided into a spatial constant term and temporal term. In particular, we presented a new position solution algorithm combining difference using the Loran system conveniently based on pseudo-range measurement. Some conclusions about this method are:

The new algorithm, not limited by the chain, is better than the traditional hyperbolic method. Besides, it is capable of decreasing the GDOP because of greater participation of the transmitters in the position solution. The repeatable accuracy could be improved without changing the amplitude of random noise.

In addition, the absolute accuracy corresponded to the measurement’s bias in the pseudo-range, such as SF and ASF. So, in the new method using difference, it is convenient to eliminate the measurement bias of the pseudo-range to improve the absolute position accuracy due to the clear error sources. In detail, absolute positioning offset decreased proportionally with the increase in correction degree about the pseudo-range. 

The work in this paper could be applied to the Loran receiver with a filter restraining random noise, thereby improving the repeatable accuracy of the Loran system further. It is able to promote the safety of HEA and the development of the Loran receiver. In the future, the Loran system may create greater value within the navigation field.

## Figures and Tables

**Table 1:** Location of sites used in the calculation.

Flag	Location	Latitude (N)	Longitude (E)
M	RongCheng	37.0644	122.3228
X	HeLong	42.7199	129.1075
Y	XuanCheng	31.0689	118.8860
Z	Raoping	23.7239	116.8958

**Table 2:** The GDOP of user sites.

Test Site	Latitude(N)	Longitude(E)	GDOP	
			Three Sites	Four Sites
A	25.000	125.000	18.476	3.721
B	35.000	130.000	4.295	2.774
C	25.000	135.000	18.292	6.946

**Table 3:** Specification of simulation parameters, *n_s_* = 1.000315.

Test Site	Transmitters	$\mathrm{Distances}\;(R_{d}/m)$	PF/μs	SF+ASF/μs	Pseudo-Range (ρ/m)
A	M	1,361,597.312	4,543.230	2.719	1,362,412.499
	X	2,001,273.422	6,677.632	4.178	2,002,526.074
	Y	901,698.633	3,008.690	1.673	902,200.037
	Z	834,208.694	2,783.497	1.521	834,664.528
B	M	728,502.840	2,430.789	1.285	728,887.962
	X	860,486.081	2,871.177	1.580	860,959.626
	Y	1,125,186.922	3,754.402	2.180	1,125,840.485
	Z	1,780,238.864	5,940.108	3.674	1,781,340.376
C	M	1,800,246.82	6,142.864	3.720	1,801,362.021
	X	2,038,381.588	6,801.451	4.263	2,039,659.613
	Y	1,718,993.171	5,735.750	3.535	1,720,052.800
	Z	1,841,004.383	6,142.864	3.813	1,842,147.447

**Table 4:** The position error’s directional components, random noise with STD 50ns.

*SF + ASF*	Number	Test Site	Lat Error Average/m	Lat Error STD/m	Log Error Average/m	Log Error STD/m
Yes	Three	A	1240.696	191.300	−1353.648	65.061
		B	381.39	29.958	−474.168	49.089
		C	270.754	154.478	−595.915	134.361
	Four	A	371.544	30.192	−1097.384	35.255
		B	576.355	15.359	−737.312	34.628
		C	304.614	48.458	−622.784	68.172
Yes ^1^	Three	A	873.035	191.346	−948.423	65.974
		B	267.893	30.632	−331.792	49.735
		C	190.103	159.826	−418.229	140.051
	Four	A	260.044	30.605	−768.590	34.547
		B	403.782	16.262	−514.944	36.179
		C	212.640	46.631	−434.992	64.4859
Yes ^2^	Three	A	370.236	185.145	−407.631	63.306
		B	115.498	30.396	−144.487	49.337
		C	81.238	162.273	−176.940	142.134
	Four	A	111.064	29.137	−328.444	33.520
		B	173.122	16.335	−222.153	36.548
		C	88.517	47.753	−184.277	67.344
No	Three	A	0.920	197.696	−2.288	67.840
		B	−0.538	29.899	1.440	50.042
		C	−10.626	150.576	10.775	133.709
	Four	A	−0.626	31.427	0.607	37.680
		B	1.262	16.771	−1.199	37.655
		C	−0.311	46.741	0.474	64.937

^1^SF+ASF is decreased 30% in pseudo-range. ^2^ SF+ASF is decreased 70% in pseudo-range.

## Appendix A. Position Solution Algorithm

The Loran is a navigation system that operates at 100 kHz. The Loran uses a hyperbolic positioning technique to estimate one’s location [34]. Then, pseudo-ranges like GNSS are developed [21].

Different from the former algorithms in [22], another processing method is presented in this paper. The pseudo-range equation is expressed as:

(A1) $$N_{i}(t)C=n_{s}R_{di}+C\left(SF_{i}+ASF_{i}+\Delta ASF_{i}\left(t\right)\right)+C\left(\delta T_{r}+P_{r}\right)+C\xi_{i}\left(t\right)$$

The algorithm is based on a GNSS-like pseudo-range solution of all available measurements using least squares. In order to describe it clearly, $C\left(SF_{i}+ASF_{i}+\Delta ASF_{i}\left(t\right)\right)$ is neglected due to its smaller amplitude, however, its influence is reflected in the measurement.

In the geodetic coordinate system, the Loran system transmitter’s position $\left(\varphi_{i},\lambda_{i}\right)$ is known, and the receiver position (φ,λ), $R_{di}$ and $\delta T_{r}+P_{r}$ are three unknown parameters to be sought. The distance between the transmitter and receiver on an ellipsoid could be calculated by many formulas. A universal formula used in the field of navigation is the Andoyer–Lambert formula [35]:

(A2) $$R_{di}=S_{0i}+\Delta S_{i}$$

where:

(A3) $$\{\begin{array}{c} S_{0i}=a\delta_{0i}\\ cos\delta_{0i}=\sin\varphi_{i}sin\varphi +cos\varphi_{i}cos\varphi cos\left(\lambda -\lambda_{i}\right)\\ \Delta S_{i}=a\frac{f}{4}\left[\frac{sin\delta_{0i}-\delta_{0i}}{1+cos\delta_{0i}}{\left(sin\varphi +sin\varphi_{i}\right)}^{2}-\frac{sin\delta_{0i}+\delta_{0i}}{1-cos\delta_{0i}}{\left(sin\varphi -sin\varphi_{i}\right)}^{2}\right] \end{array}$$

$S_{0i}$ is the arc distance of a sphere, a is the radius of the Earth, $\delta_{0i}$ is the geocentric corner between the transmitter and receiver, $\Delta S_{i}$ is a correction due to the sphere to ellipsoidal surface, f=(a−b)/a is the oblateness of the ellipsoid and a and b refer to semi-major and semi-minor axes, respectively, of the WGS-84 ellipsoid.

It is necessary to measure three pseudo-ranges at least used to find a position. So, the process is started by an assumed position $P_{0}\left(\varphi_{0},\lambda_{0},T_{u0}\right)$ and Equation (A1) had to be linearized using a Taylor expansion:

(A4) $$\{\begin{array}{c} \rho_{1}-n_{s}R_{d1}\left(\varphi_{0},\lambda_{0},T_{u0}\right)\approx \frac{\partial R_{d1}}{\partial \varphi }\Delta \varphi {|}_{P_{0}}+\frac{\partial R_{d1}}{\partial \lambda }\Delta \lambda {|}_{P_{0}}+\frac{\partial R_{d1}}{\partial T_{u}}\Delta T_{u}{|}_{P_{0}}\\ \rho_{2}-n_{s}R_{d2}\left(\varphi_{0},\lambda_{0},T_{u0}\right)\approx \frac{\partial R_{d2}}{\partial \varphi }\Delta \varphi {|}_{P_{0}}+\frac{\partial R_{d2}}{\partial \lambda }\Delta \lambda {|}_{P_{0}}+\frac{\partial R_{d2}}{\partial T_{u}}\Delta T_{u}{|}_{P_{0}}\\ \rho_{3}-n_{s}R_{d3}\left(\varphi_{0},\lambda_{0},T_{u0}\right)\approx \frac{\partial R_{d3}}{\partial \varphi }\Delta \varphi {|}_{P_{0}}+\frac{\partial R_{d3}}{\partial \lambda }\Delta \lambda {|}_{P_{0}}+\frac{\partial R_{d3}}{\partial T_{u}}\Delta T_{u}{|}_{P_{0}} \end{array}$$

where $\rho_{i}=N_{i}C$ and $R_{di}$ represents the distance between the transmitter and the assumed position. With the help of matrix inversion, the linearized observation equation is thus given by:

(A5) A·X=B

where

(A6) $$A=\left[\begin{array}{ccc} \frac{\partial R_{d1}}{\partial \varphi } & \frac{\partial R_{d1}}{\partial \lambda } & \frac{\partial R_{d1}}{\partial T_{u}}\\ \frac{\partial R_{d2}}{\partial \varphi } & \frac{\partial R_{d2}}{\partial \lambda } & \frac{\partial R_{d2}}{\partial T_{u}}\\ \frac{\partial R_{d3}}{\partial \varphi } & \frac{\partial R_{d3}}{\partial \lambda } & \frac{\partial R_{d3}}{\partial T_{u}} \end{array}\right],\;X=\left[\begin{array}{c} \Delta \varphi \\ \Delta \lambda \\ \Delta T_{u} \end{array}\right],B=\left[\begin{array}{c} \rho_{1}-n_{s}R_{d1}\left(\varphi_{0},\lambda_{0},T_{u0}\right)\\ \rho_{2}-n_{s}R_{d2}\left(\varphi_{0},\lambda_{0},T_{u0}\right)\\ \rho_{3}-n_{s}R_{d3}\left(\varphi_{0},\lambda_{0},T_{u0}\right) \end{array}\right]$$

The most important process is to get the partial derivatives in matrix A.

(A7) $$\frac{\partial R_{di}}{\partial \varphi }=a\frac{\partial \delta_{0i}}{\partial \varphi }+a\frac{\partial \Delta \delta_{i}}{\partial \varphi }$$

where

(A8) $$\frac{\partial \delta_{0i}}{\partial \varphi }=\frac{\cos\varphi_{i}\sin\varphi \cos\left(\lambda -\lambda_{i}\right)-\sin\varphi_{i}\cos\varphi }{\sqrt{1-{\left[\sin\varphi_{i}\sin\varphi +\cos\varphi_{i}\cos\varphi \cos\left(\lambda -\lambda_{i}\right)\right]}^{2}}}$$

However, $\Delta \delta_{i}$ is very complex, which is a function of $\delta_{0i}$, φ and λ. The partial differential of φ is given by:

(A9) $$\frac{\partial \Delta \delta_{i}}{\partial \varphi }=\frac{\partial \Delta \delta_{i}}{\partial \delta_{0i}}\cdot \frac{\partial \delta_{0i}}{\partial \varphi }+\frac{\partial \Delta \delta_{i}}{\partial \varphi }$$

The right part is expressed:

(A10) $$\{\begin{array}{c} \begin{array}{c} \frac{\partial \Delta \delta_{i}}{\partial \delta_{0i}}=\frac{f}{4}\left[\frac{\left[\left(cos\delta_{0i}-1\right)\left(1+cos\delta_{0i}\right)+\left(sin\delta_{0i}-\delta_{0i}\right)sin\delta_{0i}\right]\cdot {\left(sin\varphi +sin\varphi_{i}\right)}^{2}}{{\left(1+cos\delta_{0i}\right)}^{2}}\right]-\\ \frac{f}{4}\left[\frac{\left[\left(cos\delta_{0i}+1\right)\left(1-cos\delta_{0i}\right)-\left(sin\delta_{0i}+\delta_{0i}\right)sin\delta_{0i}\right]\cdot {\left(sin\varphi -sin\varphi_{i}\right)}^{2}}{{\left(1-cos\delta_{0i}\right)}^{2}}\right] \end{array}\\ \frac{\partial \Delta \delta_{i}}{\partial \varphi }=\frac{f}{4}\left[\frac{\left(sin\delta_{0i}-\delta_{0i}\right)\left[2\left(sin\varphi +sin\varphi_{i}\right)\right]cos\varphi }{1+cos\delta_{0i}}\right]-\frac{f}{4}\left[\frac{\left(sin\delta_{0i}+\delta_{0i}\right)\left[2\left(sin\varphi -sin\varphi_{i}\right)\right]cos\varphi }{1-cos\delta_{0i}}\right] \end{array}$$

Besides,

(A11) $$\frac{\partial R_{di}}{\partial \lambda }=a\frac{\partial \delta_{0i}}{\partial \lambda }+a\frac{\partial \Delta \delta_{i}}{\partial \lambda }$$

where

(A12) $$\frac{\partial \delta_{0i}}{\partial \lambda }=\frac{\cos\varphi_{i}\cos\varphi \sin\left(\lambda -\lambda_{i}\right)}{\sqrt{1-{\left[\sin\varphi_{i}\sin\varphi +\cos\varphi_{i}\cos\varphi \cos\left(\lambda -\lambda_{i}\right)\right]}^{2}}}$$

Similarly, the partial differential of $\Delta \delta_{i}\;about$ λ is given by:

(A13) $$\frac{\partial \Delta \delta_{i}}{\partial \lambda }=\frac{\partial \Delta \delta_{i}}{\partial \delta_{0i}}\cdot \frac{\partial \delta_{0i}}{\partial \lambda }+\frac{\partial \Delta \delta_{i}}{\partial \lambda }$$

Now, all components in matrix A are listed in Equations (A5) and (A6). Based on the expression of the matrix, the least squares solution is derived as:

(A14) $$X={\left(A^{T}\cdot A\right)}^{-1}\cdot A^{T}\cdot B$$

The assumed position $P_{0}\left(\varphi_{0},\lambda_{0},T_{u0}\right)$ is updated by the increment $\left(\Delta \varphi ,\Delta \lambda ,\Delta T_{u}\right)$ and the process is repeated. If the new update is less than 1mm, then no further iterations are considered necessary and arrive at a “best” Loran fix. The whole process of position solution based on pseudo-ranges is clear. The accuracy of the derived position solution can be calculated by comparing the ground truth fixes.
